# Bandpass sorting of heterogeneous cells using a single surface acoustic wave transducer pair

**DOI:** 10.1063/5.0040181

**Published:** 2021-01-27

**Authors:** Gergely Simon, Caroline Busch, Marco A. B. Andrade, Julien Reboud, Jonathan M. Cooper, Marc P. Y. Desmulliez, Mathis O. Riehle, Anne L. Bernassau

**Affiliations:** 1 School of Engineering and Physical Sciences, Heriot-Watt University, Edinburgh EH14 4AS, United Kingdom; 2 Institute of Molecular Cell and Systems Biology, Centre for Cell Engineering, University of Glasgow, Glasgow G12 8QQ, United Kingdom; 3 Institute of Physics, University of São Paulo, São Paulo 05508-090, Brazil; 4 James Watt School of Engineering, University of Glasgow, Glasgow G12 8LT, United Kingdom

## Abstract

Separation and sorting of biological entities (viruses, bacteria, and cells) is a critical step in any microfluidic lab-on-a-chip device. Acoustofluidics platforms have demonstrated their ability to use physical characteristics of cells to perform label-free separation. Bandpass-type sorting methods of medium-sized entities from a mixture have been presented using acoustic techniques; however, they require multiple transducers, lack support for various target populations, can be sensitive to flow variations, or have not been verified for continuous flow sorting of biological cells. To our knowledge, this paper presents the first acoustic bandpass method that overcomes all these limitations and presents an inherently reconfigurable technique with a single transducer pair for stable continuous flow sorting of blood cells. The sorting method is first demonstrated for polystyrene particles of sizes 6, 10, and 14.5 *μ*m in diameter with measured purity and efficiency coefficients above 75 ± 6% and 85 ± 9%, respectively. The sorting strategy was further validated in the separation of red blood cells from white blood cells and 1 *μ*m polystyrene particles with 78 ± 8% efficiency and 74 ± 6% purity, respectively, at a flow rate of at least 1 *μ*l/min, enabling to process finger prick blood samples within minutes.

## I. INTRODUCTION

Lab-on-a-chip technologies have gained widespread interest over the past two decades for their capabilities in shrinking the size of complex laboratory setups to a few square centimeters,^1,2^ thereby reducing the required amount of reagents, speeding up processing time and promising portable and minimally invasive medical diagnosis.^3,4^

Sample preparation is often required to detect low levels of biomarkers in diagnostic samples, involving processes such as separation, enrichment, or sorting of particles, cells, and biomolecules. Not only does the increase of the purity of samples enable a higher sensitivity of detection, but in the case of blood, almost any of its constituents, if isolated properly, holds diagnostic information. Cell-free plasma has been used for early cancer detection.^5,6^ Nucleated red blood cells (RBCs) can indicate anemia and are used to monitor the status of a fetus.^7^ White blood cells’ genetic information provides an ability to assess genetic diseases,^8,9^ and the isolation of circulating tumor cells (CTCs) has been shown to aid in tailoring cancer treatment and prognostic^10^ as well as reduce cancer metastasis through filtration.^11–14^ For therapeutic use, stem cells can be extracted for tissue engineering^15^ and purified blood platelets can be used for transfusion during surgery.^16^

Various active and passive techniques are available for the manipulation of cells, bacteria, or other biological entities.^17–26^ Passive methods use specific geometrical features within the device to achieve separation without any external field and are, therefore, usually fixed for a given application and cannot be adapted to suit other or evolving target populations.^17,18^ Active techniques use external fields and are generally reconfigurable for various target populations unless they exhibit nonlinear phenomena.^19–26^ Sorting can be carried out by the size, density, polarizability, or magnetic properties of the micro-objects under consideration, and a contactless and label-free nature is required to avoid the alteration of cell function or induction of cell necrosis.^19^

Among all sorting methods, acoustic techniques offer the capability of fast sorting based on cell size,^20–22^ density,^23,24^ or compressibility,^25^ while maintaining cell viability,^26^ without the need for any cell markers.^27^ They are therefore well suited for reconfigurable, continuous flow label-free separation and are usually differentiated by the transducer configuration and the employed electrical signal. Most commonly used are standing wave sorters either in bulk acoustic wave (BAW) or surface acoustic wave (SAW) devices.^20,25,28,29^ They achieve sorting based on a time-of-flight principle and, therefore, are highly sensitive to flow rate variations and require fine tuning of acoustic energy density as well as tight thermal control (within 1 °C) achieved via Peltier cooling.^30^ Selective marking with microbubbles can allow even similar sized entities to be sorted.^31^ Traveling wave sorters can be more selective as they utilize a nonlinear frequency dependence of acoustic radiation force but for the same reason lose generic reconfigurability.^21,32,33^ The combination of traveling and standing waves for sorting has been successfully used for flow rate insensitive sorting, but the adaptability is not well discussed.^22,34^ Dynamic methods, such as employing phase or frequency modulation, can be an alternative for stable sorting against flow rate variations, while maintaining reconfigurability.^35–37^

Bandpass sorting, enabling to filter or retain particles or cells between two threshold values, is increasingly important for cell mixtures, such as blood, where the heterogeneity of the sample is more pronounced. For example, a passive flow fraction-based filter device was investigated by Kim *et al.* to trap and thus sort living cells between 18 and 30 *μ*m in diameter.^38^ Acoustic bandpass sorting can be achieved using a standing wave sorter by adjusting the flow rate and acoustic energy density, either by having multiple outlet channels or cascading single sorting stages. A multiple outlet configuration allows for sorting particles of different sizes into defined outlets, as demonstrated by Petersson *et al.*^28^ A cascaded device was presented by Skowronek *et al.*,^39^ whereby two stages of standard time-of-flight devices allowed to sort medium-sized entities. However, as discussed for time-of-flight standing wave sorters, both configurations suffer from flow rate sensitivity. To overcome this issue, Ma *et al.* exploited the nonlinear frequency dependence of the acoustic contrast factor in traveling waves by using two different frequencies.^40^ However, the devices cannot be reconfigured for arbitrary sorting scenarios with the same efficiency as they are fixed by design for specific size ranges. Further improvement of multistage sorting can be achieved by cascading a traveling wave stage after a standing wave stage, as demonstrated by Wang *et al.*^41^ Theoretically, even either a tilted angle standing wave sorter^25^ or an attenuated traveling wave pair could be used for bandpass sorting,^22^ but these articles do not discuss such type of target extraction and the corresponding efficiencies.

In this paper, we present a simple acoustic device built from a single pair of ultrasonic transducers driven by reconfigurable acoustic bandpass waveforms for improved sorting of a wide population of particles based on their sizes or mechanical properties. This active, dynamic method combines conventional surface acoustic wave transducer and modulated acoustic standing waves, allowing to generate a tailored acoustic radiation force that accurately selects the particles of interest out of a wide range of particles or cells mixture without any restriction of particle size. The method has also good stability against flow rate variations as discussed before^42^ and therefore offers a simple and stable way of adaptive extraction of target cells in a continuous flow with minimal driving components. We demonstrate the potential of our tailored waveform acoustic technique for sorting mixtures of synthetic polystyrene (PS) particles and further apply it to complex biological cells mixtures.

## II. SORTING WORKING PRINCIPLE

**FIG. 1.**
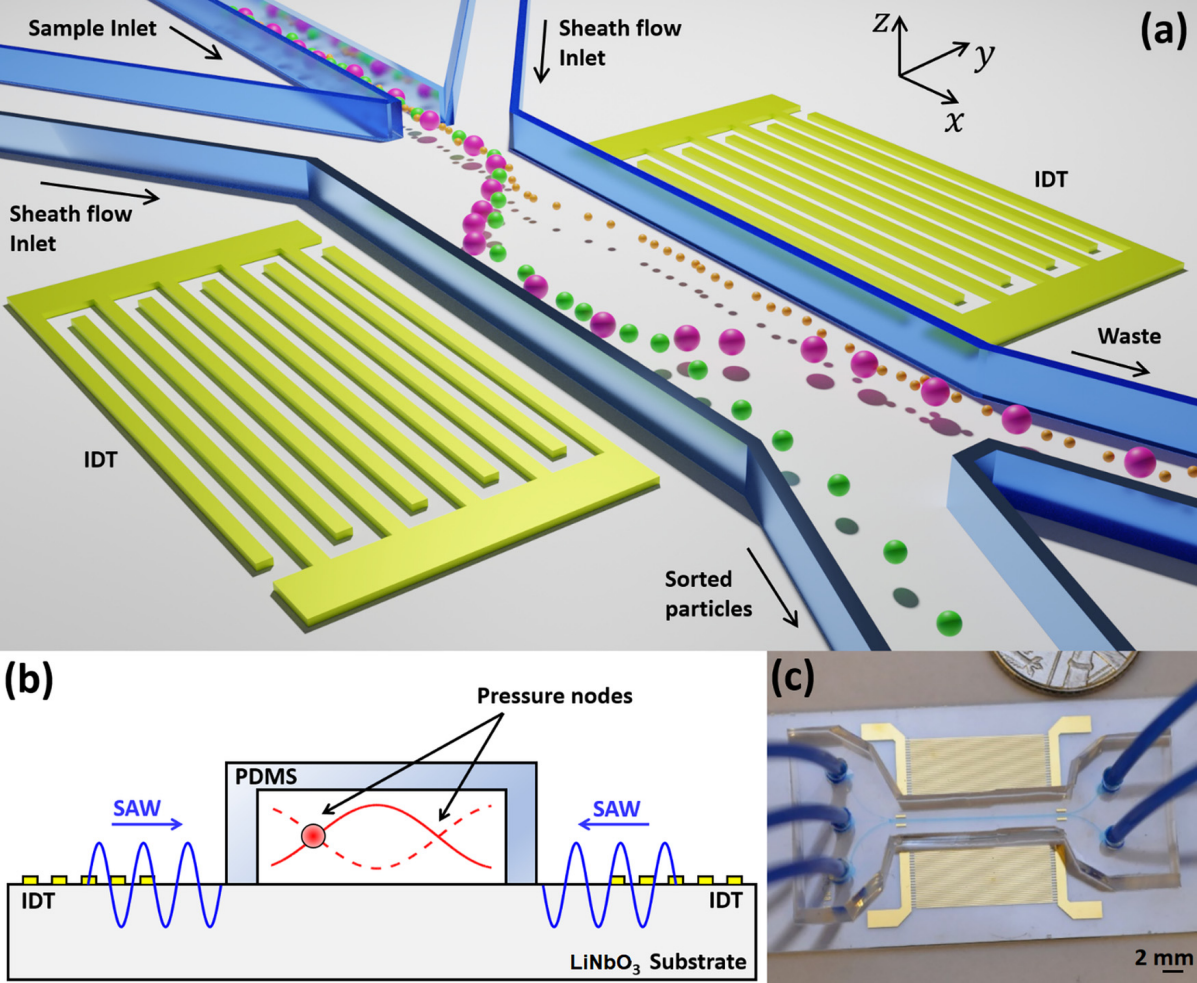
(a) Schematic of the bandpass acoustofluidic device. A mixture of particles of different sizes is injected through the middle inlet. Middle-sized particles (green) are channeled to the sorted particles outlet after separation from the smaller (orange) and larger (purple) particles. The inlet configuration is asymmetric: the sample inlet is 50, while the two sheath inlets 50 and 140 *μ*m wide. (b) Cross-sectional image of the device, emphasizing the bulk acoustic standing wave that traps and translates micro-objects. (c) Photograph of the sorting device. Sorted particles exit at either the bottom (B) or top outlet (T). These sorting setups are referred to as downward and upward sorting, respectively.

The bandpass acoustofluidic device is depicted in Fig. 1. The device is similar to a configuration employed by others,^25,38^ with the difference that we use asymmetric trifurcated inlets instead of a symmetric configuration. A particle or cell mixture is injected through the middle inlet, while a sheath flow is provided through both side inlets [Fig. 1(a)].

When the particle mixture enters the main channel, its initial transverse position, *y*, can be controlled by adjusting the flow rates of both sheath flows.^37^ By applying sinusoidal electrical excitation on the electrodes of the interdigital transducers (IDTs), surface acoustic waves (SAWs) are generated through the inverse piezoelectric effect and propagate toward the fluidic channel. Here, the waves refract into the fluid contained in the cavity, and the interference of the two head-on waves create standing waves in the transverse direction, trapping particles and cells at the pressure nodes due to the action of the acoustic radiation force [Fig. 1(b)].^43^ By controlling the standing wave pattern slowly in time in a deterministic and dynamic manner, as a result of either phase^44^ or frequency modulation^37^ of the signals applied to each IDT, particles of a chosen, desired size (or of desired mechanical properties) are separated and exit the device through the sorted particles outlet, whereas the rejected particles leave through the waste channel [Fig. 1(a)]. The fabricated acoustofluidic device is shown in Fig. 1(c). For further information on the fundamentals of frequency modulated sorting and theoretical discussion, see the supplementary information of Ref. 37.

### A. Single transducer bandpass sorting

**FIG. 2.**
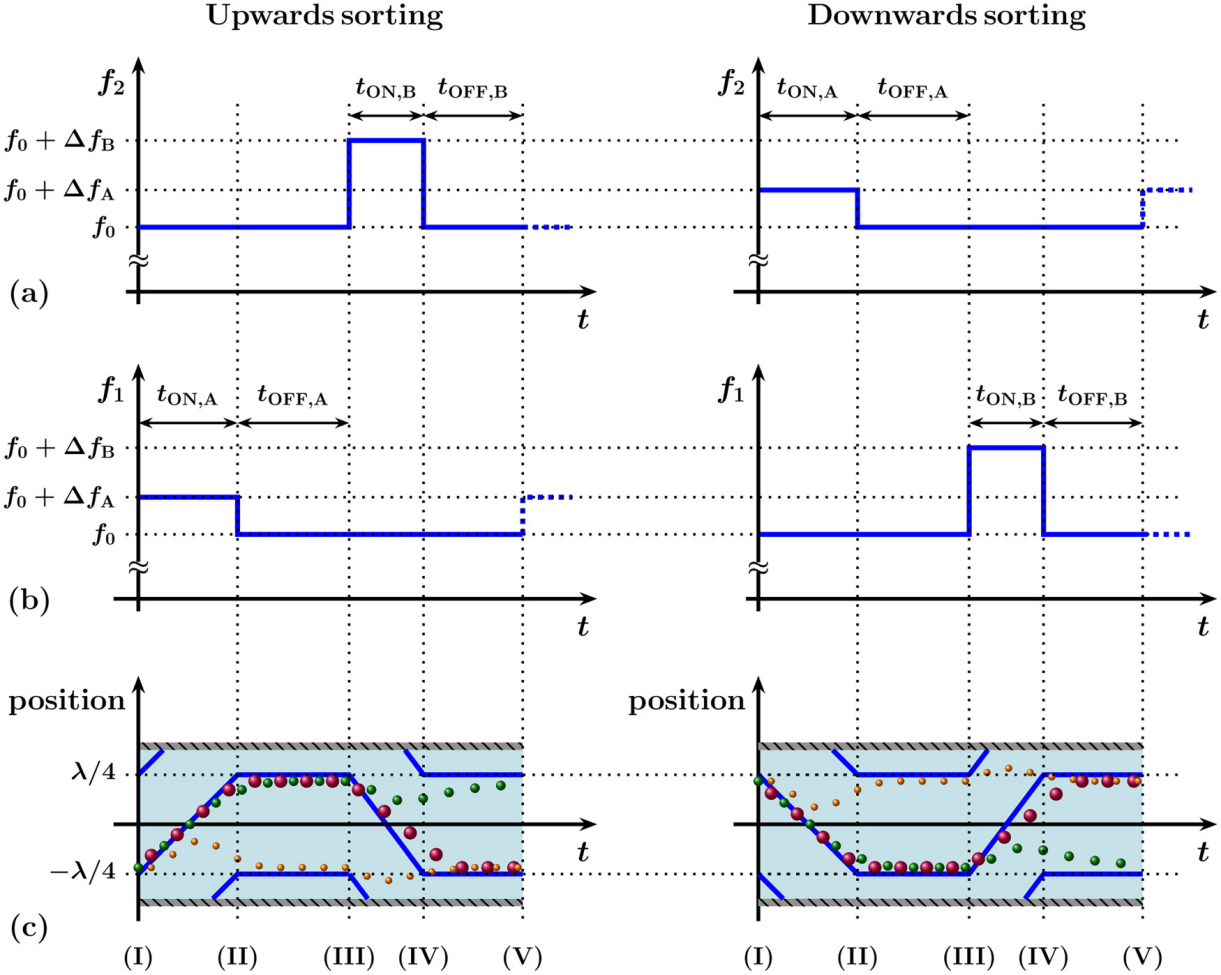
Coupling two modulation cycles directly allows for performing bandpass sorting of the middle size particles in a three-size particles mixture. (a) Frequency pattern of the top electrode. (b) Frequency pattern of the bottom electrode. (c) Resulting movement of the pressure nodes (blue thick lines) and resulting particle trajectories (orange small, green middle, and red large particles). Refer to the supplementary document of Ref. 37 for theoretical details and verification of pressure node movement.

In this work, we demonstrate a new implementation of our dynamic sorting method to achieve “bandpass sorting,” where a middle-sized particle is selected and sorted from a set of particles containing both smaller and larger particles (Fig. 2).

In the “upward” sorting case (left column in Fig. 2), the targeted, medium-sized particles exit the device at the top outlet. In the initial step, all particles are focused at the trapping node, located at −λ/4, as shown in Fig. 2(c)—I. During the first stage of sorting referred to as I to III on the timeline, the larger-sized particles (10 and 14.5 *μ*m nominal size) relax at the other pressure node, the sorting node located at λ/4. During the second stage of sorting, from III to V, by adjusting the frequency difference, only the largest particles are moved back toward the original trapping node, leaving the middle-sized particles at the sorting node.^37^ The reverse “downward” sorting (right column) results in the target particles exiting at the bottom outlet [as also shown in Fig. 1(a)]. The second sorting stage can be achieved either by an adjusted frequency difference, keeping the pressure (voltage) constant ( $\Delta f_{A}\neq \Delta f_{B}$ and $p_{0,A}=p_{0,B}$) or by adjusting the acoustic pressure within the channel (via the transducer voltage) and keeping the frequency difference constant ( $p_{0,A}\neq p_{0,B}$ and $\Delta f_{A}=\Delta f_{B}$). For brevity, these two modes of operation are referred to as CVBP sorting (constant voltage bandpass sorting) and CFDBP sorting (constant frequency difference bandpass sorting). The CVBP approach is straightforward and easy to perform by the control software enabling easier design, while more precision is available with the second approach. Note that typical frequency differences between transducers are less than 1 Hz, which is 7 orders of magnitude less than the usual ∼10 MHz drive frequencies. Even a narrow band transducer would allow for these adjustments without significant change in impedance and performance. Finally, as the timeline suggests, the flow speed should be adjusted to allow for a whole two stage separation. Slower flow speeds, where additional (partial) sorting stages can occur within the timeline, only shift the non-target particles closer to the channel wall and do not affect target extraction.

## III. MATERIALS AND METHODS

### A. Theoretical analysis

When a spherical particle of radius a flows into the region between the IDTs, the standing wave field generates a lateral acoustic radiation force on the particle,^45–47^ given by^43^

$$F_{\mathrm{ac}}=-\frac{4\pi a^{3}}{3}\frac{u_{0}^{2}\rho_{0}}{4}k\left[\;f_{1,\kappa }+\frac{3}{2}f_{2,\rho }\right]\;\sin(2ky)\hat{y}=-V_{p}E_{\mathrm{ac}}k\Phi_{\mathrm{ac}}\;\sin(2ky)\hat{y},$$ (1)

with

$$f_{1,\kappa }=1-\kappa_{p}/\kappa_{0},$$ (2a)

$$f_{2,\rho }=\frac{2(\rho_{p}/\rho_{0}-1)}{2\rho_{p}/\rho_{0}+1},$$ (2b)

$$\Phi_{\mathrm{ac}}=\left[\;f_{1,\kappa }+\frac{3}{2}f_{2,\rho }\right],$$ (2c)

where y is the particle position along the *y* axis [Fig. 1(a)], κ and ρ are compressibility and density, respectively, and the index *p* denotes a particle property and 0 a fluid property. In Eq. (1), $V_{p}=4\pi a^{3}/3$ is the particle volume, *k* is the wavenumber, and $E_{\mathrm{ac}}=u_{0}^{2}\rho_{0}/4=p_{0}^{2}\kappa_{0}/4$ is the energy density, where $p_{0}$ is the acoustic pressure amplitude. The compressibility- and density-dependent terms can be grouped together to obtain the acoustic contrast factor, $\Phi_{\mathrm{ac}}$. The material properties of both cells and particles used in this paper result in a positive acoustic contrast factor. Therefore, cells and particles are trapped at the acoustic pressure nodes.

### B. Time-of-flight sorting

For the simplest standing wave sorting devices, also called time-of-flight separation devices, the only force to balance the radiation force is the hydrodynamic drag force, proportional to the particle radius,

$$F_{\mathrm{drag}}=-6\eta \pi a\dot{y}=-c_{\mathrm{visc}}\dot{y},$$ (3)

where η is the dynamic fluid viscosity and $\dot{y}$ is the velocity of the particle. The theoretical trajectories of particles can be derived for modulated standing wave devices from the force balance equation such that^37^

$$y(t)=\frac{\Delta \omega t}{2k}-\frac{1}{k}\;\mathrm{ta}n^{-1}\left[\frac{\gamma -Q\;\tan((c_{1}-t)Q/2)}{\Delta \omega }\right],$$ (4)

with $Q=\sqrt{{\left(\Delta \omega \right)}^{2}-\gamma^{2}}$ and $\gamma =2kc_{ac}/c_{visc}$. The term *c*_ac_ is $V_{p}E_{\mathrm{ac}}k\Phi_{ac}$ and the constant $c_{1}$ is found through the initial position of the particle. An expression for the limit, when a particle is located at the antinode, results in a transcendental equation and the numerical solution,

γ/Δω≈4.2503, (5)

which can be used to choose the appropriate timing and frequency difference, once γ is known,

$$\gamma \propto a^{2}\Phi_{ac}\Rightarrow 1/\Delta \omega \propto a^{2}\Phi_{ac},$$ (6)

This scaling law^37^ can be used to adjust the sorting to various particle populations within a single device.

The bandpass sorting is carried out as described in Sec. II A, and particle trajectories can be generated by stitching two sets of trajectories together as shown in this section. For the CVBP sorting, Δω=2πΔf is adjusted between the two cycles, while for CFDBP sorting, the acoustic energy density is varied (via the acoustic pressure and voltage).

### C. Device design

The operating wavelength was chosen to be 300 *μ*m to minimize device size and enable handling of 5–20 *μ*m diameter objects, a range covering the size of wide range mammalian cells, for example. This corresponds to a frequency of 13.3 MHz on the lithium niobate 128°-Y cut wafer (substrate velocity $c_{\mathrm{sub}}=3990\;\;ms^{-1}$). The channel was 50 *μ*m in height and its width was 240 *μ*m. This allows two pressure nodes to fit within the device (λ/2 = 150 *μ*m), and on both sides of the channel, a safety margin of 45 *μ*m was left for the anechoic corner.^48,49^ The channel was fabricated in PDMS (polydimethylsiloxane) using standard soft photolithography techniques.^44^

### D. Experimental setup

The device [ca. 40 × 20 mm—Fig. 1(c)] was mounted on a 100 × 100 mm printed circuit board with a square opening to allow for visual inspection. Flow was provided using syringe pumps (World Precision Instruments, Sarasota, USA), while the electrical excitation originated from a signal generator (TG5012A, Aim-TTi, UK) controlled via USB connection, from a PC. The signal was amplified (ZHL-1-2W+, Mini-Circuits, UK) before being delivered to the transducers.

The particle and cell behaviors were investigated using an upright brightfield optical microscope (Olympus BX51, Olympus, UK). Particle trajectories were recorded at 80 fps using a black and white camera (Orca Flash 2.8, Hamamatsu, UK).

### E. Cell preparation

Human T lymphocytes (Jurkat, ATCC) were cultured using RPMI 1640 (Fischer Scientific UK) supplemented with 10% fetal bovine serum (Fischer Scientific UK) and a mixture of penicillin and streptomycin antibiotics (Fischer Scientific UK). Red blood cells were obtained from a whole blood finger prick sample from a healthy volunteer who provided informed consent.

### F. Acoustic cell characterization

Direct and indirect methods are available to assess the acoustic contrast factor of biological cells. Precise measurements of the cell density and compressibility, followed by calculation of the contrast factor as per Eq. (2), provide a direct approach. Alternatively, reference particles can be used and the cell behavior (trajectories) can be monitored while being subjected to an acoustic field, and thus obtaining the contrast factor indirectly.^50,51^ This method can be further simplified: according to the acoustic–viscous drag force balance,^43^ different particles or cells have different maximum speeds that are proportional to their size and acoustic contrast factor so that

$$v_{\max}=\frac{c_{\mathrm{ac}}}{c_{\mathrm{visc}}}\propto a^{2}\Phi_{\mathrm{ac}}.$$ (7)

Therefore, after measurement of the particle and cell size, the maximum slope of the trajectories (that gives maximum speed) can be used to calculate the unknown contrast factor,

$$\Phi_{\mathrm{ac}}=\frac{a_{\mathrm{ref}}^{2}}{a^{2}}\frac{v_{\max}}{v_{\max,\mathrm{ref}}}\Phi_{\mathrm{ac},\mathrm{ref}},$$ (8)

where the subscript ref denotes the properties of the reference particle. The advantages of this method compared to the trajectory fitting are as follows: (i) no need to fit trajectories one-by-one, (ii) no need to calibrate for different acoustic pressure amplitudes from experiment to experiment, and (iii) no need to calibrate for offset errors in the trajectories.

## IV. RESULTS AND DISCUSSION

### A. Device characterization

**FIG. 3.**
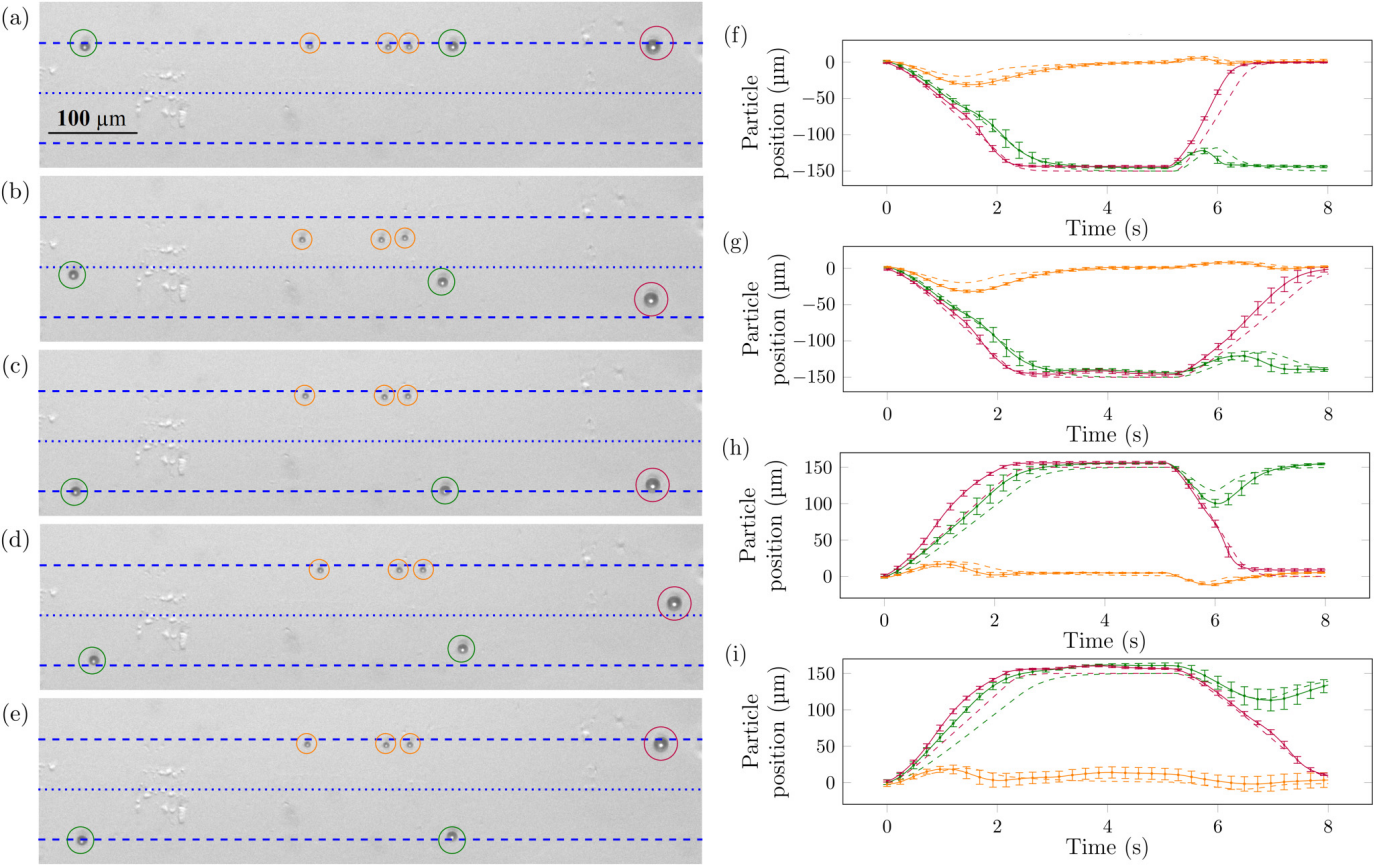
Bandpass sorting of synthetic 6 *μ*m (orange circles and lines), 10 *μ*m (green circles and lines), and 14.5 *μ*m (purple circles and lines) PS particles. (a)–(e) Downward bandpass sorting, video frames. Dashed blue lines indicate the position of the pressure nodes where particles trap; the dotted blue line is the pressure antinode, corresponding time points are 0, 2.0, 4.5, 7.0, and 8.0 s. (f)–(g) Experimental (solid lines) and analytical (broken lines) sorting curves for upward bandpass sorting particle trajectories using (f) fixed voltage sorting, CVBP, and (g) fixed frequency difference sorting, CFDBP. (h)–(i) Experimental (solid lines) and analytical (broken lines) sorting curves for downward bandpass sorting particle trajectories for (h) CVBP and (i) CFDBP sorting. Videos SV1–SV4 are available in the supplementary material.

The feasibility of the downward bandpass sorting method was first tested in the absence of flow using polystyrene (PS) particles of 6, 10, and 14.5 *μ*m diameter, Fig. 3. Before sorting, all particles locate at the top pressure node where they are trapped by the primary acoustic radiation force [Fig. 3(a)]. After the first frequency modulation stage, the two larger-sized spheres (10 and 14.5 *μ*m) locate on the bottom side of the pressure antinode [Fig. 3(b) (note—in top views, bottom refers to the right-hand side of the channel when considering a flow direction from left-to-right)]. After the first full frequency modulation/resting stage, the two largest spheres are trapped at the bottom pressure node, while the small particles positioned themselves at the top pressure node [Fig. 3(c)]. After the second frequency modulation stage, spheres of the largest size are pushed back toward the top (left side on top view considering the left to right flow) and cross the pressure antinode again, while the medium-sized spheres only get displaced by an amount too small to allow them to cross the antinodal line [Fig. 3(d)]. After the full sorting cycle, the smallest and largest spheres locate themselves at the top pressure node, while the medium-sized 10 *μ*m particles are sorted at the bottom pressure node [Fig. 3(e)].

The particle trajectories were measured and compared using fixed voltage and fixed frequency difference methods [Figs. 3(f)–3(i)]. The resting time was 3 s in all cases. Using the scaling law in Eq. (6), the voltage required to achieve sorting in the second stage with the same 0.46 Hz frequency difference is 16.3 Vpp, the value that was used in the experiments. The switching of the voltage occurs at the middle of the resting phase, small “bumps” are visible where the voltage of the transducers is adjusted (these are also noticeable in the supplementary material videos). This is due to the serial switching of the two transducers; they momentarily have unbalanced voltages thus delivering non-uniform acoustic energy to the channel making the particles translate briefly. Apart from this behavior, the two approaches (CVBP and CFDBP sorting) performed similarly (see supplementary material videos). For simplicity, the constant voltage bandpass sorting method was subsequently used in the next experiments.

**FIG. 4.**
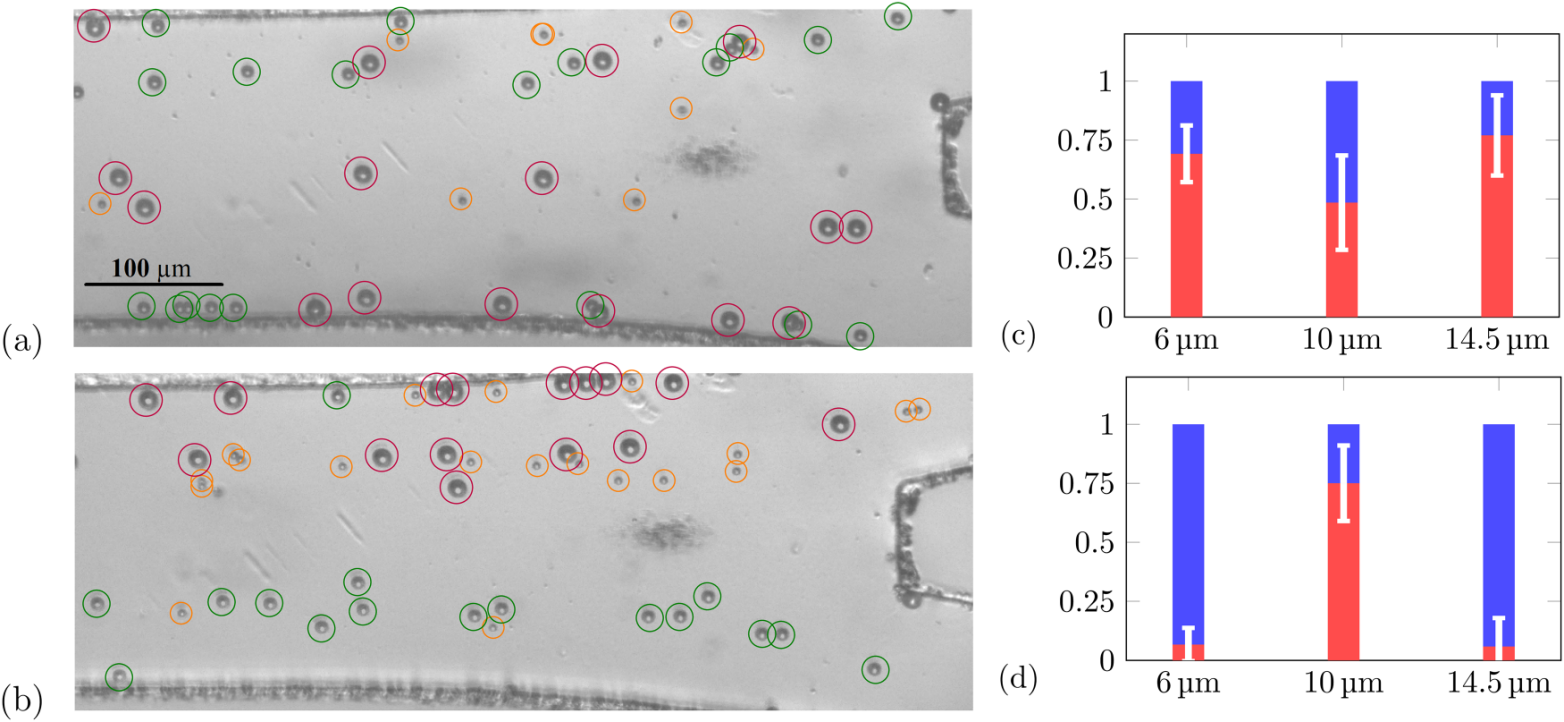
Bandpass sorting of 6, 10, and 14.5 *μ*m PS particles. Overlay images recorded at the device outlet for (a) upward sorting and (b) downward sorting. (c) and (d) Quality of bandpass sorting of 6, 10, and 14.5 *μ*m PS particles. Blue and red portions illustrate particles going to the top outlet and bottom outlet, respectively, upward (c) and downward sorting (d). Error bars correspond to standard deviation of five experiments.

By adding flow (0.4, 0.15, and 0.5 *μ*l/min for the three inlets) to the acoustic excitation, we demonstrated continuous flow bandpass sorting as shown in Fig. 4. The average normalized efficiency^42^ is 51 ± 8% and 75 ± 6% for the upward and downward methods, respectively, while the average normalized purity^42^ is 49 ± 11% and 85 ± 9%, illustrating the superiority of the downward sorting method. This is possibly due to transducer differences due to manufacturing imperfections and the asymmetric inlet configuration.

As Refs. 36 and 37 suggest, the method can be applied to sort particles based on material property difference, such as density.

**FIG. 5.**
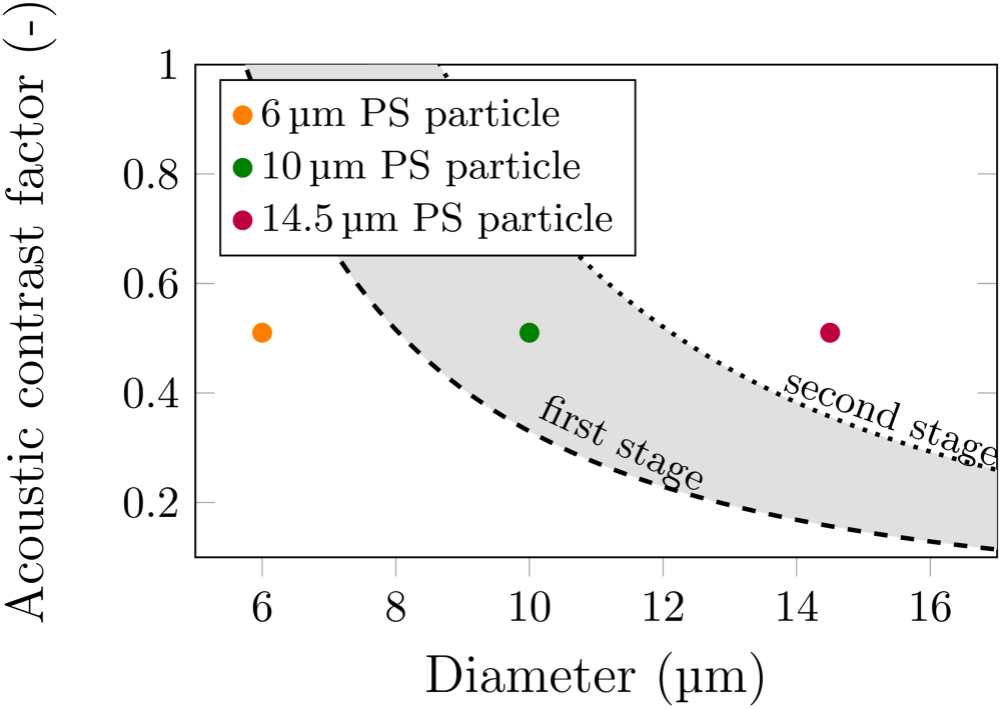
Visual illustration of the choice of the sorting parameters and the limits of the sorting for bandpass separation of 6, 10, and 14.5 *μ*m PS particles. The broken and dotted lines correspond to the first and second sorting stages, respectively. During the first stage, the particles above the inverse-square dashed line get displaced, which are the 10 and 14.5 *μ*m ones. During the second stage, only the largest 14.5 *μ*m particle gets sorted (above the dotted line), leaving behind the 10 *μ*m ones as illustrated by the gray area between the dashed and dotted lines.

Using the asymmetric inlet configuration, particles can be focused easier toward the wall closer to the center inlet. This naturally supports downward sorting. The sorting of different particle populations can be visualized graphically utilizing the limit of sorting given by Eq. (5) and the scaling law provided by Eq. (6) as shown in Fig. 5.

### B. Cell sorting experiments

**FIG. 6.**
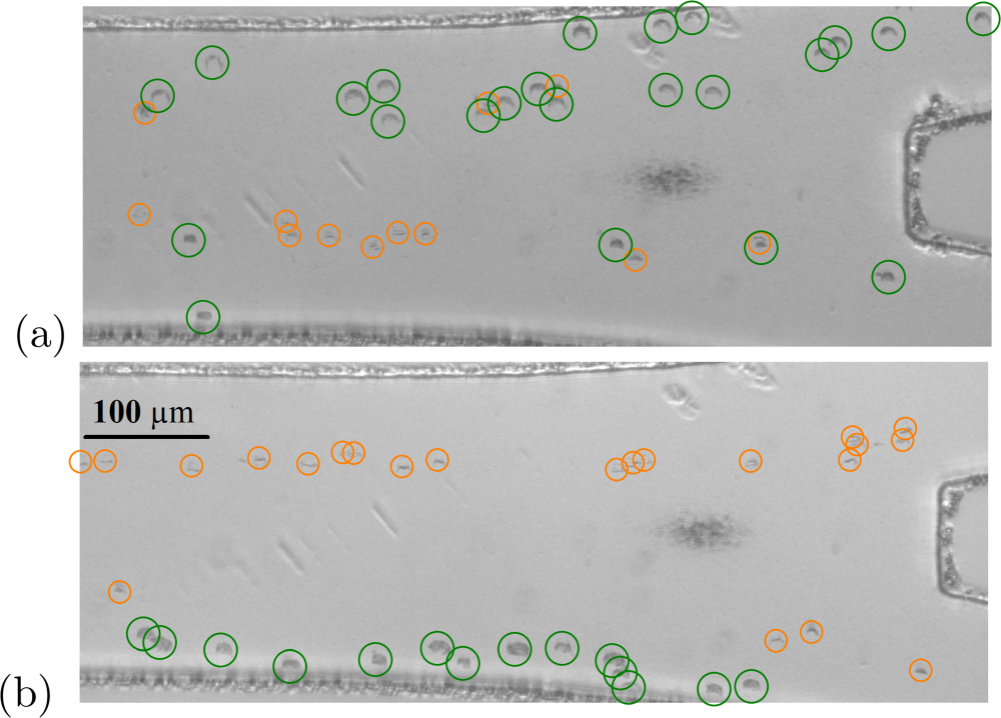
Separation of red blood cells (orange circles) and white blood cells (green circles) for (a) upward direction where the sorted white blood cells exit at the top and (b) downward direction where the sorted white blood cells exit at the bottom. Videos SV5 and SV6 are available in the supplementary material.

The performance of the device was characterized by sorting RBCs and Jurkat cells representing white blood cells as shown in Fig. 6. Furthermore, Table I provides the parameters, along with the values of the figures of merit.

**TABLE I:** Sorting parameters and figures of merit for separation of red blood cells and white blood cells. Flow 1 and flow 2 are the two sheath volumetric flow rates.

Direction of travel	Flow 1 (*μ*l/min)	Flow 2 (*μ*l/min)	Flow 3 (*μ*l/min)	Frequency (Hz)	Rest time (s)	Efficiency (%)	Purity (%)
Downward	0.2	0.23	0.7	0.85	3	94 ± 3	84 ± 6
Upward	0.8	0.25	0.2	0.80	3	88 ± 3	93 ± 5

**FIG. 7.**
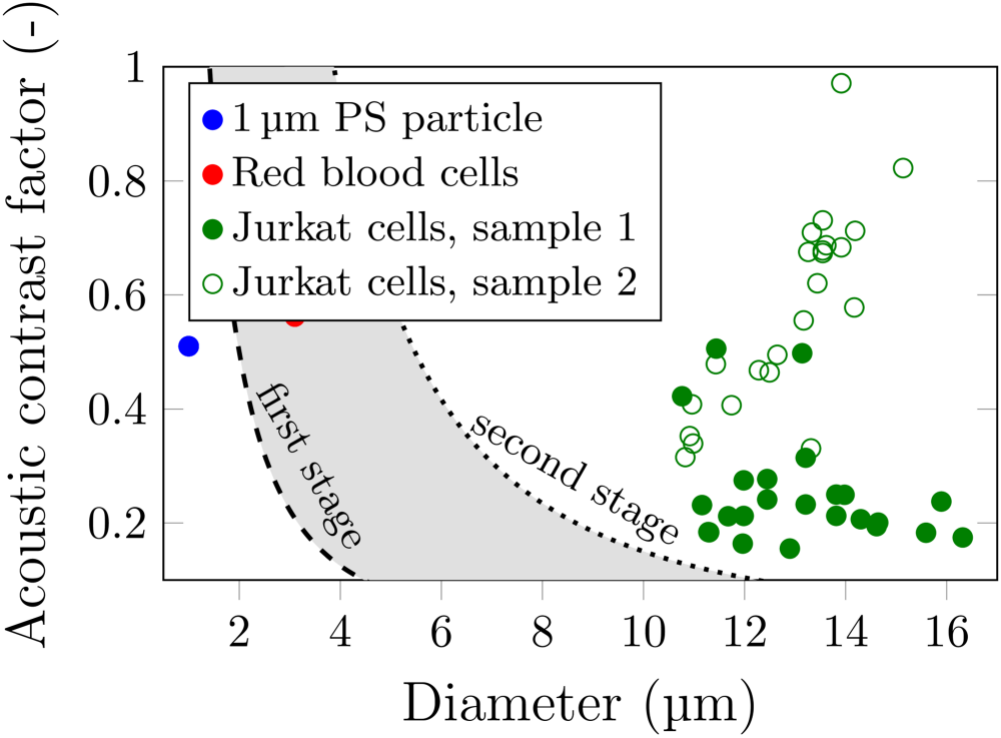
Visual illustration of the choice of the sorting parameters and the limits of the sorting for bandpass separation of red blood cells from 1 *μ*m PS particles and white blood cells. The 1 *μ*m PS particles are used as surrogates for debris or platelets. Two random samples of Jurkats were investigated; these are marked as sample 1 and sample 2. Their contrast factor difference is attributed to changes in life cycle.

The contrast factor for the red blood cells was obtained from Eq. (2) and values from the literature,^52^ while for the white blood cells, a more rigorous investigation was carried out following the methodology described in Sec. III F, as it is less readily available in the literature. The results are plotted in Fig. 7 illustrating a wider spread of both size and contrast factor.

The results still allowed the fitting of the two sorting curves between the particles and cells demonstrating thereby bandpass separation of the red blood cells. As with the PS particles, the first stage separates both types of cells from 1 *μ*m particles, and the second stage enables the white blood cells to return to their original location, leaving the medium-sized red blood cells sorted.

**FIG. 8.**
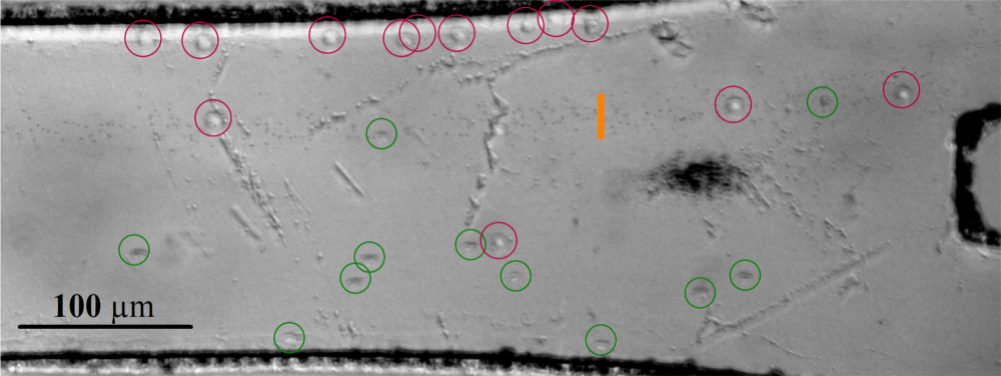
Bandpass separation of 1 *μ*m PS particles (orange line indicating spread), RBCs (green circles), and Jurkat cells (purple circles). The input voltage, 27 Vpp, was applied on the transducers, with frequency modulation parameters of 0.4 Hz and 0.6 Hz during the two cycles, with 0.5 s and 1 s off times, respectively. Flow rates were set at 0.3 *μ*l/min, 0.2 *μ*l/min, and 0.5 *μ*l/min for the top, middle, and bottom outlet, respectively.

Bandpass separation experiments of particles showed an even more significant imbalance between downward and upward sorting than for the single two-particle experiments.^37^ Therefore, for cell sorting, the focus was placed solely on downward bandpass sorting as this method shows the highest efficiency. 1 *μ*m PS particles were used to mimic platelets or small cell fragments (debris). Figure 8 is where the target RBCs, circled in green, exit at the bottom outlet. The spread of non-target 1 *μ*m PS particles is indicated by an orange line, while the non-target Jurkat cells are circled in red. The separation has an overall efficiency of 78 ± 8% and 74 ± 6% purity.

## V. CONCLUSIONS

We investigated the reconfigurable, continuous flow bandpass sorting with a single pair of transducer for selecting particles and cells of interest. First, we presented the analytical description of the method, discussed the scaling laws, and compared the two bandpass techniques (CVBP or CFDBP) for particle sorting applications. During the entire separation, either the transducer voltage (and consequently acoustic pressure, CVBP) or the frequency difference (CFDBP) between the two transducers was kept constant. The consequences of these two sorting techniques in terms of particles or cells trajectories were presented. Furthermore, the direction of sorting (the waste and target output channels) can be swapped as illustrated for particle mixtures. Continuous flow sorting of 6, 10, and 14.5 *μ*m diameter particles was carried out with 1.05 *μ*l/min flow rate and showed ∼50% efficiency and purity for the upward sorting direction, and 75% efficiency and 85% purity for the downward sorting direction. Due to the asymmetric inlet configuration, the efficiency and purity of the experiments also showed asymmetric behavior: the particle focusing efficiency is better toward the channel wall that is closer to the trapping node used. This, in turn, results in better figures of merit for downward sorting.

For biological cells, continuous flow sorting was performed for red blood cells and white blood cells, with 1 *μ*m polystyrene particles used as surrogates for blood platelets and debris. Using the superior downward sorting direction, for 1 *μ*l/min total flow rate, we observed 78 ± 8% efficiency and 74 ± 6% purity. The bandpass cell sorting technique has the potential to be applied widely in lab-on-a-chip devices due to its simplicity, sorting stability, and reconfigurability.

## SUPPLEMENTARY MATERIAL

Supplementary material videos are available online. Videos SV1–SV4 show CVBP and CFDBP sorting methods for particles, both in the downward and upward direction. Videos SV5 and SV6 illustrate the continuous flow separation of red blood cells and white blood cells.

## Data Availability

The data that support the findings of this study are available from the corresponding author upon reasonable request.
